# Male Infertility and Reduced Life Expectancy: Epidemiology, Mechanisms, and Clinical Implications

**DOI:** 10.3390/jcm14113930

**Published:** 2025-06-03

**Authors:** Aris Kaltsas, Andreas Koumenis, Marios Stavropoulos, Zisis Kratiras, Dimitrios Deligiannis, Konstantinos Adamos, Michael Chrisofos

**Affiliations:** Third Department of Urology, Attikon University Hospital, School of Medicine, National and Kapodistrian University of Athens, 12462 Athens, Greece; ares-kaltsas@hotmail.com (A.K.); a_koumeni@icloud.com (A.K.); stamarios@yahoo.gr (M.S.); zkratiras@gmail.com (Z.K.); d.delijohn@yahoo.gr (D.D.); constantinos.adamos@gmail.com (K.A.)

**Keywords:** male infertility, life expectancy, cardiometabolic risk, psychosocial factors, hypogonadism, cancer risk, men’s health

## Abstract

Male infertility is a prevalent condition affecting approximately 15% of couples worldwide. Recent evidence indicates that, beyond its immediate reproductive implications, male infertility may reflect broader health concerns. Large-scale cohort studies consistently show that men with poorer semen parameters have elevated all-cause mortality compared to fertile counterparts, with a dose-dependent pattern whereby more severe abnormalities correlate with a higher risk of early death. Proposed mechanisms linking infertility to reduced life expectancy encompass genetic, hormonal, and lifestyle factors. For instance, Klinefelter syndrome exemplifies a genetic cause of azoospermia that also predisposes to metabolic syndrome, diabetes, and certain malignancies. Low testosterone, a frequent finding in testicular dysfunction, is implicated in obesity, insulin resistance, and cardiovascular disease, all of which can shorten lifespan. Additionally, psychosocial stress and depression—commonly reported among infertile men—may contribute to health-compromising behaviors. Environmental exposures and socioeconomic factors further compound these risks. Collectively, these data underscore the importance of recognizing male infertility as an early indicator of potentially modifiable health vulnerabilities. A comprehensive evaluation of infertile men should therefore extend beyond fertility assessments to include screening for chronic diseases, hormonal imbalances, and mental health issues. Targeted surveillance for specific cancers (e.g., testicular and prostate) and early interventions—such as lifestyle modifications, appropriate hormonal therapies, and psychosocial support—can improve both reproductive outcomes and long-term well-being. Given these insights, male fertility assessment may serve as a valuable gateway to broader men’s healthcare, prompting proactive strategies that mitigate associated risks and potentially enhance longevity.

## 1. Introduction

Infertility, defined as the inability to conceive after 12 months of regular unprotected intercourse, is a prevalent global health concern affecting approximately 15% of couples, with male factors contributing to nearly half of these cases [1,2]. Historically, research and clinical management of male infertility have predominantly addressed reproductive outcomes; however, emerging evidence suggests that male reproductive health reflects broader systemic health, potentially influencing overall longevity. Therefore, male infertility may represent an early clinical biomarker indicative of general health status and potential life expectancy [3,4].

The scope of this narrative review extends beyond the immediate reproductive implications of infertility, emphasizing its significance as a predictor of chronic disease and premature mortality. Growing epidemiological evidence demonstrates a noteworthy association between infertility and higher prevalence rates of chronic conditions such as malignancies, cardiovascular disease, and metabolic disorders among infertile men compared to fertile counterparts [5,6]. These findings suggest shared underlying mechanisms, including genetic predispositions, developmental abnormalities, hormonal imbalances, and lifestyle factors like obesity and smoking, which could concurrently impair reproductive function and systemic health [7,8].

Despite these correlations, significant gaps persist in understanding the comprehensive nature of these associations, particularly regarding the integrated effects of psychosocial stress and its potential to exacerbate health outcomes. Men experiencing infertility often report elevated psychological distress, including higher levels of stress, depression, and anxiety, compared to fertile men [9]. Chronic psychological distress, influenced by societal expectations and perceptions surrounding masculinity and fatherhood, may indirectly increase the risk of cardiovascular, metabolic, and immunological disorders, further implicating infertility as a complex, multifaceted health concern [10].

To address these critical gaps in the literature, this review synthesizes current epidemiological data linking male infertility to reduced life expectancy, explores underlying biological mechanisms, discusses the psychosocial dimensions, and highlights potential lifestyle and environmental influences. The review structure includes: (1) a summary of epidemiological evidence establishing the link between infertility and mortality; (2) exploration of common comorbidities such as cardiovascular, metabolic, oncological, and endocrine disorders; (3) detailed discussion of biological and molecular mechanisms underlying these associations; (4) evaluation of psychosocial impacts and their health implications; and (5) recommendations for clinical practice, emphasizing a multidisciplinary approach for comprehensive men’s health management. Through this integrative perspective, the review aims to reinforce the recognition of male infertility not only as a reproductive issue but also as a crucial indicator of overall male health, informing future clinical practices and public health strategies.

## 2. Epidemiological Links Between Male Infertility and Mortality

A growing body of evidence from large population-based cohort studies and meta-analyses indicates that men with infertility face higher all-cause mortality compared to fertile counterparts. The elevated risk persists even after adjusting for baseline health, socioeconomic status, and other confounders, suggesting a true biological or systemic link rather than merely a lifestyle artifact. While the magnitude of the increase in mortality varies among studies, a consistent dose–response relationship emerges: the more severe the sperm impairment, the greater the risk of premature death.

Pioneering work by Jensen et al. (2009) in over 43,000 Danish men first suggested that semen quality correlates with longevity. They observed a steady decrease in mortality as sperm concentration, motility, and normal morphology improved, up to a threshold of about 40 million sperm per milliliter. Men with very low sperm counts suffered substantially increased mortality—largely from chronic diseases—thereby positioning semen quality as a fundamental biomarker of male health and survival [11].

Subsequent studies corroborate this link. In a U.S. cohort of nearly 12,000 men, Eisenberg et al. (2014) reported that those with multiple abnormal semen parameters had more than double the risk of death (adjusted hazard ratio [HR] ~2.3) over an eight-year follow-up, compared to men with entirely normal semen profiles [12]. More recently, a Danish cohort of ~78,000 men followed for up to 50 years showed that men with the highest total motile sperm counts (>120 million) lived on average to 80.3 years, whereas those with very low counts (<5 million) lived ~2.7 years less (77.6 years). The lowest-fertility group had a 61% higher risk of death than men in the highest-fertility group (HR ~1.61) [13]. These findings underscore a gradation of risk with declining reproductive potential.

A 2021 systematic review and meta-analysis spanning ~60,000 men found that infertile men have a 26% higher risk of all-cause mortality than fertile men (pooled HR = 1.26). Moreover, when categorizing by semen parameters, those with oligospermia or azoospermia had a 67% higher mortality risk relative to men with normal counts, and completely azoospermic men (no sperm output) faced an approximate twofold greater hazard of death compared to normospermic men [14]. This review also illustrated the “healthy patient” effect: compared to the general population, men who seek fertility care can appear healthier, but within that care-seeking group, having impaired semen parameters still confers significant excess mortality.

Another meta-analysis from 2023 quantified the hazard ratio for all-cause mortality in infertile men at ~1.37 [15]. While effect sizes vary, the consensus remains that male infertility is associated with a modest but clinically meaningful increase in the risk of dying earlier, particularly among men with severe sperm abnormalities.

Research suggests a portion of this excess mortality arises from cardiovascular disease and malignancies. Eisenberg et al. (2016) reported that infertile men were at significantly increased risk for chronic conditions including diabetes, ischemic heart disease, hypertension, renal and liver diseases, and peripheral vascular disease [16]. Similarly, a Swedish national registry study by Lundberg et al. (2019) observed that men with “infertility-related” diagnoses (e.g., varicocele, cryptorchidism) had higher mid-life mortality than controls (HR ~1.23), while those explicitly labeled “infertile” showed no overall excess compared to controls—perhaps reflecting better healthcare engagement and risk management in the latter group [17]. Notably, this same Swedish study documented an age-dependent effect: men diagnosed with infertility under age 30 experienced a threefold higher risk of death, largely attributable to cancers diagnosed near the time of fertility evaluation.

Several other cohorts highlight elevated cardiovascular risks and specific malignancies. For instance, the Malmö Preventive Project found that childless men, taken as a proxy for infertility, had a 23% higher all-cause mortality and 33% higher cardiovascular mortality compared to fathers—along with more adverse metabolic risk factors at baseline [18]. In the realm of cancer, a meta-analysis by Behboudi-Gandevani et al. (2021) identified a 43% increased overall cancer risk (odds ratio [OR] ~1.43) in infertile men versus fertile men, with especially heightened risks for testicular cancer (OR ~1.91), prostate cancer (OR ~1.48), and melanoma (OR ~1.31) [19]. Furthermore, men fathering through assisted reproductive techniques—particularly those requiring ICSI (intracytoplasmic sperm injection)—appear to face higher prostate cancer risk, including early-onset disease, as shown by Al-Jebari et al. (2019) in a cohort of over one million Swedish fathers (HR ~1.64 for all prostate cancer, and ~1.86 for diagnosis before age 55) [20].

Taken together, these findings indicate that male infertility signals broader susceptibilities, including vascular pathologies, metabolic disorders, and malignant conditions. Some of these vulnerabilities likely stem from shared etiological factors (e.g., endocrine or genetic abnormalities) that simultaneously impair spermatogenesis and elevate disease risk. Table 1 summarizes the major epidemiological studies and meta-analyses, along with their key quantitative outcomes linking male infertility to increased mortality and cancer risks.

Overall, the quantitative data show that male infertility—a marker often detected in the prime of life—heralds higher risks of mortality from cardiovascular disease, cancers, and potentially other chronic conditions. These findings underscore the importance of comprehensive health assessments in infertile men, including proactive cardiovascular screening and targeted cancer surveillance when appropriate. Rather than simply reflecting a short-term issue of reproductive concern, impaired semen parameters can serve as an early warning sign of systemic vulnerabilities that may predispose to life-limiting conditions in the decades to follow [4].

Nonetheless, it must be emphasized that these epidemiological associations do not by themselves prove a direct cause-and-effect relationship between male infertility and reduced lifespan. All evidence to date comes from observational studies, which—despite adjustments for known confounders—cannot fully eliminate the influence of other factors. Lifestyle influences, socioeconomic status, and healthcare-seeking patterns (for instance, the “healthy patient” effect) might partly account for the mortality differences observed. Therefore, caution is warranted in interpreting male infertility as a causal factor per se in early mortality; it is more appropriate to view it as a sensitive biomarker of underlying health status rather than an independent determinant of longevity [21].

Moreover, the possibility of reverse causation should be considered. Undiagnosed systemic diseases or incipient pathologies could impair a man’s fertility while also contributing to premature mortality, thus confounding the link between infertility and lifespan. In such scenarios, infertility is less the cause of subsequent health problems and more an early symptom of an underlying condition that ultimately drives risk. For example, chronic illnesses like latent cardiovascular disease, diabetes, or even early-stage malignancies might first manifest subtly by disrupting spermatogenesis long before overt clinical signs appear. Recognizing this possibility underscores why infertile men should undergo thorough health evaluations—not because infertility itself directly shortens life, but because it may unmask broader health vulnerabilities that, if addressed early, can improve both fertility and long-term health outcomes [21].

Epidemiological evidence continues to mount that male infertility is not only associated with immediate reproductive challenges but also foreshadows long-term health risks. Several recent large-scale cohort studies with extended follow-up have bolstered this link. For example, a Danish registry study tracking ~4700 men for up to three decades found that those with severely impaired semen parameters had significantly higher rates of later cardiovascular disease and metabolic illnesses compared to men with normal fertility [22]. Crucially, these associations persisted even after rigorous adjustment for key confounders–including age, body mass index, smoking status, socioeconomic indicators, and baseline health–suggesting that male infertility is an independent predictor of future morbidity [23]. Likewise, a population-based cohort (mean follow-up ~18 years) observed that childless men (as a proxy for infertility) bore a greater midlife burden of metabolic syndrome and type 2 diabetes than fathers [24]. Taken together, these methodologically robust studies (with careful control of confounders and >10-year follow-ups) underscore the notion that poor male reproductive health can serve as an early warning sign of elevated long-term risks, from cardiometabolic disease to reduced longevity [22,23].

## 3. Common Comorbidities in Infertile Men

In recent years, researchers have identified several common comorbidities and health risks that tend to cluster with male infertility. Importantly, not all infertility cases carry the same degree of risk—differences in underlying phenotype or etiology appear to modulate the comorbidity profile. For instance, men with non-obstructive azoospermia (NOA), reflecting intrinsic testicular failure, often exhibit greater systemic morbidity than those with obstructive azoospermia (OA) where spermatogenesis is intact. NOA is frequently accompanied by hypogonadism, a hormonal disturbance linked to metabolic syndrome and cardiovascular disease [25]. Consistent with this, men with NOA have higher rates of chronic illnesses (e.g., diabetes and heart disease) and an elevated mortality risk relative to fertile men [26]. By contrast, infertility due to purely obstructive causes is not associated with testicular endocrine dysfunction, and these men may not incur the same cardiometabolic risk elevation. In support of this divergence, a large cohort study observed that only men with the most severe spermatogenic impairment (azoospermia) had a significantly increased risk of early death, whereas men with milder infertility (oligospermia or unexplained factor) showed no significant mortality difference from fertile controls [27].

Meanwhile, idiopathic infertility represents a heterogeneous group whose overall health prognosis is less clearly defined. Epidemiological data suggest that idiopathic or mild male-factor infertility portends a comparatively lower increase in long-term disease risk than the above severe or genetic forms [27]. Indeed, one study found no significant mortality difference for men with idiopathic oligospermia versus fertile counterparts [27]. Nonetheless, even idiopathic infertile men show modest elevations in certain chronic conditions when compared to fertile men, indicating that any fertility impairment may signal some degree of health vulnerability [26]. Recognizing these phenotype-specific risk differences is important for tailoring clinical evaluation and counseling–men with high-risk infertility etiologies (e.g., NOA due to genetic causes) warrant especially vigilant health monitoring, whereas those with isolated obstructive or mild idiopathic infertility may require a more general but still comprehensive health assessment.

### 3.1. Cardiovascular Disease and Metabolic Syndrome

A consistent finding is that infertile men have higher rates of cardiovascular risk factors. In a large Stanford study of ~9387 men with fertility issues (median age 38), 44% had at least one other significant health problem apart from their reproductive complaint. The most notable associations were with diseases of the circulatory system–hypertension, peripheral vascular disease, and heart disease were substantially more common in men with poor semen quality. In fact, this was the first time such a link had been shown, highlighting a surprising overlap between reproductive health and cardiovascular health [28]. Subsequent studies have corroborated these findings. A 2018 Swedish registry study by Elenkov et al. examined men who became fathers via intracytoplasmic sperm injection (ICSI) (typically used for severe male-factor infertility) versus men who conceived naturally. The ICSI fathers had a 15% higher risk of requiring medication for hypertension (HR 1.15) and a 28% higher risk of receiving medications for metabolic syndrome (a combination of drugs for diabetes, high blood pressure, and dyslipidemia; HR 1.28) compared to naturally conceiving fathers [18]. These differences emerged within the first decade after reproduction.

Metabolic syndrome, a cluster of conditions including hypertension, hyperglycemia, dyslipidemia, and central obesity, has also been increasingly linked to male infertility. The association between metabolic syndrome and infertility is multifaceted, involving hormonal imbalances, oxidative stress, and inflammation. Infertile men often exhibit signs of metabolic syndrome, which can exacerbate their reproductive issues and contribute to broader health concerns. A nationwide analysis focused on men who needed donor sperm to have a child (a proxy for extreme spermatogenic failure such as non-obstructive azoospermia). These men exhibited significantly elevated rates of new-onset diabetes, hyperlipidemia, and low testosterone in the years after becoming fathers, relative to men who did not use donor sperm [29]. The link between infertility and cardiometabolic illness may stem in part from shared hormonal disturbances–for example, infertile men are more likely to be hypogonadal, and low testosterone is a known contributor to insulin resistance, visceral adiposity, and cardiovascular risk. Indeed, low testosterone itself has been associated with increased mortality in men, suggesting one pathway by which testicular failure (leading to androgen deficiency) could elevate long-term health risks [30]. In aggregate, these studies establish that infertile men have higher prevalence of hypertension, metabolic syndrome, and heart disease, identifying male infertility as a potential early warning sign of future cardiometabolic problems [31].

Moreover, recent meta-analytic data (2023) strengthen these observations by indicating that men with infertility face approximately a 39% higher hazard of developing type 2 diabetes and about a 20% higher risk of major cardiovascular events compared to fertile peers. In a 30-year follow-up, the incidence of type 2 diabetes reached 25% in infertile men, versus ~17% in controls [17]. Hypogonadism frequently underlies these cardiometabolic vulnerabilities, potentially exacerbating insulin resistance and increasing the likelihood of metabolic syndrome and heart disease [32]. These insights highlight the importance of early identification and management of metabolic risk factors in men presenting with infertility.

### 3.2. Oncologic Concerns

One of the most significant health concerns in infertile men is an elevated risk of certain cancers. Testicular cancer and prostate cancer–both male-specific malignancies–have been closely linked with male infertility. Infertility and testis cancer share etiologic ties (for example, cryptorchidism in early life increases risk of both conditions), and prostate cancer in later life has been hypothesized to be more frequent among men with impaired fertility. Recent evidence bears this out. A comprehensive 2021 meta-analysis reported that infertile men have nearly double the odds of developing testicular cancer compared to fertile men (pooled odds ratio [OR] ~1.91, 95% confidence interval [CI] 1.52–2.42). The same analysis found a 48% increase in risk of prostate cancer among men with a history of infertility (pooled OR ~1.48, 95% CI 1.05–2.08). These findings align with earlier cohort studies [19]. For example, Walsh et al. and others have shown that men evaluated for infertility had a higher incidence of testicular germ cell tumors subsequently than expected, suggesting a screening benefit in this population [33].

Regarding prostate cancer, a landmark Swedish registry study (Al-Jebari et al., 2019) tracked over 1.1 million fathers and stratified them by mode of conception [20]. Men who conceived via in vitro fertilization (IVF) or ICSI had a significantly higher hazard of prostate cancer than men who conceived naturally, even at relatively young ages. Notably, fathers who used ICSI–typically the most severe male-factor cases–had a 64% higher risk of prostate cancer overall (HR 1.64) and nearly double the risk of early-onset prostate cancer (diagnosed before age 55, HR ~1.86) compared to natural conception fathers. IVF fathers also had an elevated risk, though not as high as the ICSI group. Moreover, the prostate cancers in the ICSI group tended to be of similar advanced stage (as inferred by use of androgen-deprivation therapy) as those in the general population, indicating that the increased screening these men undergo (by virtue of being in healthcare systems for fertility) does not fully explain the risk. Taken together, these findings suggest a genuine pathophysiological link between male infertility and malignancy [20]. Possible explanations include shared developmental risk factors (e.g., impaired androgen signaling in utero contributing to both infertility and cancer propensity) or common genetic vulnerabilities. Beyond testis and prostate cancer, some studies have also noted higher rates of melanoma and certain hematologic cancers in infertile men, though evidence is less consistent.

The meta-analysis by Behboudi-Gandevani et al. found a modest association with melanoma (pooled OR ~1.3) and specifically highlighted a higher risk of non-Hodgkin lymphoma in infertile men in one large study [19]. While these links require further confirmation, the overall cancer risk profile of infertile men points to the need for vigilance: infertility should prompt consideration of appropriate cancer screenings (such as regular testicular exams and possibly earlier prostate-specific antigen [PSA] testing in select cases). Indeed, some experts now advocate that men with infertility, particularly those with severe cases or known risk factors (e.g., prior cryptorchidism), undergo proactive cancer surveillance as part of their long-term care [19].

In addition, recent analyses indicate that infertile men not only have a roughly twofold higher risk of testicular cancer but also about a 1.6-fold higher risk of prostate cancer and a ~1.3-fold increased risk of melanoma [17]. A 2025 genetic study provides further evidence of shared susceptibility: infertile men were found to carry nearly five times as many pathogenic germline variants in known cancer-risk genes compared to fertile controls (6.9% vs. 1.5%) [34]. This enrichment of cancer-linked mutations suggests a broader genetic vulnerability that can manifest as both infertility and malignancy. Overall, these updated findings underscore that male infertility may signal significant oncologic and endocrine risks, warranting increased surveillance and patient counseling.

### 3.3. Endocrine and Autoimmune Disorders

Infertile men commonly manifest endocrine abnormalities, either as causes or consequences of their condition. Apart from hypogonadism (low testosterone), disorders such as thyroid dysfunction and pituitary abnormalities (for example, prolactinomas) can present with infertility in men and likewise carry systemic health implications [35]. Additionally, as noted in the Stanford study, men with multiple sperm defects had higher odds of skin diseases and endocrine disorders than fertile men. Some endocrine comorbidities might be directly related (e.g., congenital adrenal hyperplasia can cause infertility and adrenal issues), whereas others might reflect broader autoimmune processes or genetic syndromes [28]. As mentioned before, diabetes mellitus is another endocrine/metabolic condition frequently observed in infertile men, partly due to the overlap with metabolic syndrome. Chronic uncontrolled diabetes can impair spermatogenesis (via hormonal changes and oxidative stress) and, conversely, some causes of infertility (like hypogonadism) predispose to diabetes–creating a vicious cycle of morbidity [36]. Autoimmune diseases have also been studied: for instance, celiac disease and other autoimmune conditions are slightly more prevalent in men with infertility in some cohorts, though data are limited [37]. There is emerging interest in the role of systemic inflammation and autoimmunity (like anti-sperm antibodies or testicular autoimmunity) as both a cause of infertility and a contributor to other organ damage, but clear clinical associations (e.g., infertility with rheumatoid arthritis or lupus risk) remain to be fully defined [38].

### 3.4. Genetic Syndromes

A subset of male infertility is attributable to underlying genetic abnormalities, which often come with significant comorbidities and reduced life expectancy in their own right. The most prominent example is Klinefelter syndrome (47,XXY), the most common chromosomal cause of male infertility [39]. Klinefelter syndrome affects approximately 1 in 600 newborn males and typically causes azoospermia and testosterone deficiency. Men with Klinefelter syndrome have well-documented health risks: they are prone to metabolic syndrome, type 2 diabetes, thrombosis, osteoporosis, and certain cancers (such as breast cancer and non-Hodgkin lymphoma), all of which contribute to higher mortality [40]. Epidemiological studies indicate that Klinefelter patients have a significantly increased risk of death–roughly 40–50% higher than male population norms [41]. One analysis found a hazard ratio of about 1.4 for all-cause mortality in Klinefelter syndrome, particularly due to diabetes and cardiovascular diseases [42]. Many Klinefelter men are on lifelong testosterone replacement, which mitigates some symptoms but does not completely normalize their health risks. It is notable that Klinefelter syndrome accounts for up to 10–15% of cases of azoospermia–thus a substantial fraction of infertile men have this genetic condition driving both their infertility and comorbid health issues [43]. Other genetic causes of infertility include Y chromosome microdeletions (which primarily affect spermatogenesis genes but generally have no known impact on somatic health), cystic fibrosis transmembrane regulator (CFTR) mutations leading to congenital absence of the vas deferens (CBAVD), and various rare syndromes (for example, Kallmann syndrome of isolated gonadotropin-releasing hormone deficiency, which can feature osteoporosis and metabolic issues if not treated) [25]. CFTR mutations causing male infertility (absent vas deferens) often indicate carrier status or mild cystic fibrosis; these men can have subclinical pancreatic or pulmonary issues and are at risk for chronic sinus/lung problems, which could influence health outcomes. Another genetic consideration is the 47,XYY karyotype, which is less common but has been associated with higher risk of learning disabilities and a slightly increased risk of mortality and health issues, though data are sparse [44]. In sum, when male infertility is due to an identifiable genetic disorder, that disorder frequently brings its own spectrum of comorbidities that can decrease life expectancy. Genetic evaluation of infertile men (karyotype, Y deletion testing) is therefore not only relevant for fertility treatment but also for prognostic counseling about health.

### 3.5. Other Health Issues

Beyond the categories above, studies have noted that infertile men tend to have a greater burden of general medical illnesses. For instance, some research finds higher rates of chronic renal and liver diseases among infertile men, possibly related to metabolic syndrome or medication exposures [45]. Infertility has also been linked to an increased incidence of hospitalizations for various conditions and greater use of prescription medications in longitudinal follow-up [46]. An analysis of Swedish health registries found that men who needed assisted reproduction had higher subsequent usage of medications for hypertension, diabetes, and hyperlipidemia, as mentioned, but also showed trends toward more prescriptions overall–implying more doctor visits and diagnoses in general for these men [47].

## 4. Biological and Hormonal Mechanisms Connecting Infertility with Systemic Disease

Understanding why infertile men face increased health risks requires examining the biological mechanisms that might connect reproductive function with other physiological systems. Several interrelated mechanisms have been proposed.

### 4.1. Genetic Pleiotropy and Developmental Pathways

A compelling explanation is that many of the genes and developmental pathways that govern fertility in men also influence other aspects of physiology. It is estimated that approximately 15% of all genes in the human genome are involved in reproduction (spermatogenesis, hormone production, etc.) [48]. Evolutionarily, these genes tend to have multiple roles (pleiotropic effects). For example, a gene required for sperm production might also be important in cell cycle regulation or metabolism in other tissues [49]. Eisenberg noted that most genes related to reproduction have diverse functions beyond the gonads [50]. Therefore, a deleterious mutation or expression change that impairs fertility could concurrently affect the heart, brain, endocrine glands, or tumor suppression, to name a few possibilities [51]. This genetic pleiotropy is exemplified by conditions like Klinefelter syndrome as discussed, but even at the subclinical level, there may be genomic instability or polymorphisms that predispose a man both to infertility and to other diseases [52]. Another concept is the Testicular Dysgenesis Syndrome (TDS) hypothesis, which posits that disrupted fetal development of the testes (due to genetic or environmental factors) can lead to a suite of problems: impaired spermatogenesis (infertility), cryptorchidism, hypospadias, and higher risk of testicular cancer [53]. TDS highlights how an early developmental insult (e.g., inadequate androgen exposure in utero) can simultaneously set the stage for infertility and other health issues like cancer. It’s conceivable that developmental factors that compromise testis function also subtly affect development of other organs (for example, the vasculature or pancreas), leading to later-life disease. In summary, shared genetic and developmental factors likely underlie many of the observed links–infertility is not an isolated defect but rather one manifestation of a broader biological phenotype [54].

### 4.2. Hormonal Imbalances

Hormonal imbalances offer a direct mechanistic bridge between reproductive function and systemic health. The testes are not only sperm factories but also endocrine organs (producing testosterone, inhibin, etc.). When testicular function is impaired (as in primary testicular failure), testosterone levels often decline, and levels of pituitary hormones (luteinizing hormone [LH] and follicle-stimulating hormone [FSH]) rise in compensation. Low testosterone (hypogonadism) has well-documented effects on men’s health: it contributes to increased body fat, reduced muscle mass, insulin resistance, dyslipidemia, and endothelial dysfunction [55]. Over time, these changes elevate the risk of type 2 diabetes, atherosclerosis, and osteoporosis, among other conditions. Chronic low testosterone may therefore be one mechanism linking infertility to reduced life expectancy. Conversely, some men have infertility due to central (hypothalamic) or pituitary dysfunction (hypogonadotropic hypogonadism), which might coexist with other hypothalamic-pituitary disorders affecting adrenal or thyroid function, again impacting general health [56]. On the other hand, excess of gonadotropins in primary testicular failure could have their own effects; high FSH has been speculated to influence bone density or cognitive function, though clear evidence is lacking. Apart from gonadal hormones, hormonal treatments or exposures can also tie fertility to health [57]. For example, anabolic steroid abuse causes temporary infertility by suppressing the gonadotropin axis–it also has severe health consequences (hyperlipidemia, liver damage, cardiac stress). While such cases are a smaller subset, they illustrate how interfering with the reproductive hormone axis can have system-wide effects. Ensuring hormonal balance (through treatments like testosterone therapy when appropriate) may thus be an important strategy to improve long-term health in infertile men, though care must be taken since exogenous testosterone can impair fertility–a clinical management dilemma [58].

### 4.3. Shared Risk Factors and Organ Reserve

It is likely that some common risk factors damage both fertility and other organ systems simultaneously. For instance, systemic illnesses–even subclinical–can reduce a man’s fertility. Conditions such as obesity, poorly controlled diabetes, severe infections, or chronic inflammatory diseases (like inflammatory bowel disease) can impair spermatogenesis via increased oxidative stress or fever or cytokine effects [59]. These same conditions obviously have direct effects on health and longevity. Thus, an infertile man may be essentially a man with an underlying health issue that has not yet been diagnosed, and the fertility problem is the first manifesting symptom. One scenario is a man with undetected Type 2 diabetes or metabolic syndrome: he may present with infertility (due to erectile dysfunction, impaired spermatogenesis from obesity, or low testosterone from insulin resistance)–if one only treats the infertility without recognizing the metabolic disease, the man remains at high risk for a heart attack or stroke later [60]. Similarly, a man with an occult malignancy might present with infertility (e.g., testicular cancer often causes low sperm counts even in the early stages [61]; Hodgkin lymphoma can present with fertility issues due to fevers or immune factors) [62]. Identifying the fertility issue can sometimes lead to the diagnosis of the systemic disease–for example, fertility testing might reveal azoospermia due to obstructive azoospermia, prompting a cystic fibrosis gene test and thereby uncovering that the man is a CF carrier. In terms of organ reserve, some experts theorize that fertility is a “luxury function”–the body will sacrifice reproductive capacity in times of stress or poor health to maintain vital organs [63]. Chronic stress, severe malnutrition, or advanced chronic illness can shut down the reproductive axis (as seen in very ill patients or endurance athletes). While many infertile men are otherwise well, this concept underscores that the reproductive system is sensitive to overall health. If a man’s fertility is compromised, it may indicate that his body has, in a sense, signaled trouble–either through direct pathophysiology or as a trade-off in resource allocation [16].

### 4.4. Molecular and Cellular Mechanisms

On a finer level, there are intriguing hypotheses about cellular aging and quality control that might link infertility with systemic aging. For instance, telomere length in somatic cells has been associated with aging and lifespan; some studies have explored telomeres in sperm and testes, with mixed results, but it’s possible that men with infertility have underlying accelerated cellular aging that affects multiple tissues [64]. Mitochondrial function is another consideration: sperm production and function are highly energy-dependent, so mitochondrial dysfunction (whether due to genetic variants or accumulated damage) can cause poor sperm quality and also contribute to diseases like neurodegeneration or cardiomyopathy [65].

Oxidative stress has emerged as a pivotal molecular link between male reproductive dysfunction and systemic illness, as ROS-driven damage underlies not only sperm dysfunction but also pathologies such as inflammation, atherosclerosis, and aging [66]. At the cellular level, excessive ROS in the male reproductive tract induces peroxidative damage to the sperm’s plasma membrane and oxidative DNA fragmentation, triggering apoptotic cascades that ultimately impair sperm motility and viability [67]. Consistent with these mechanisms, infertile men often show elevated oxidative stress biomarkers–for example, higher ROS generation and lipid peroxidation coupled with diminished antioxidant capacity–compared to fertile controls [68]. A recent review emphasized that these biomarkers, including advanced oxidation protein products (AOPP), malondialdehyde (MDA), and total antioxidant capacity (TAC), may serve as diagnostic or prognostic indicators in evaluating male infertility, particularly when oxidative stress is a central etiological factor [69]. Such redox imbalances may also contribute to the long-term health risks in this population, since oxidative stress is a known driver of cardiometabolic and neurodegenerative diseases; for instance, obese subfertile men exhibit increased ROS levels and sperm DNA fragmentation alongside reduced antioxidant defenses [70], highlighting a shared oxidative pathology linking male infertility with chronic disease outcomes [71,72].

Additionally, defects in DNA repair or cell-cycle regulation might simultaneously lead to infertility (by causing meiotic errors or impaired spermatogenesis) and predispose to cancer (through accumulated mutations) [73]. Another molecular link is hormone receptors: androgen receptor (AR) gene variations can cause mild androgen insensitivity, presenting as oligospermia and subtle metabolic issues; these same AR variants could influence prostate cancer risk [74]. Disruption of key intracellular signaling cascades in spermatozoa—such as those involving tyrosine phosphorylation and calcium flux—has also been implicated in impaired motility and fertilization potential, further highlighting the role of molecular deregulation in male infertility [75]. A literature review conducted by Puzuka et al. supports the notion that genetic variations and genome instability are critical factors in the relationship between male infertility and cancer risks [76]. As these genetic factors may predispose infertile men to develop testicular cancer, thorough evaluation and regular follow-up are warranted.

Epigenetic factors deserve mention as well–environmental influences can cause epigenetic changes that affect gene expression in both reproductive and somatic cells. For instance, endocrine disruptors (discussed below) may epigenetically reprogram certain pathways leading to both low sperm counts and higher fat accumulation [77].

In summary, accumulating evidence suggests that the long-term health implications of male infertility vary depending on the underlying biological mechanism. Genetic causes—such as Klinefelter syndrome or mutations affecting DNA repair—are associated with heightened risks for metabolic disease, malignancy, and premature mortality. In contrast, hormonal dysfunctions like hypogonadism are closely linked with cardiometabolic conditions due to their direct impact on body composition, insulin sensitivity, and vascular function. Infertility related to inflammation or oxidative stress, often seen in varicocele or infections, is increasingly recognized as a systemic pro-inflammatory state that may elevate cardiovascular and metabolic risks. These mechanistic distinctions help explain why infertile men are not a homogenous population and highlight the need for tailored clinical risk assessment based on etiology [21].

## 5. Psychosocial Impact of Infertility on Mental Health

Beyond the purely biological connections, psychosocial factors play a significant role in the health of infertile men. The diagnosis of infertility can be emotionally devastating and is often accompanied by feelings of shame, inadequacy, depression, and anxiety. These mental health challenges can indirectly influence physical health and longevity. Recent studies and reviews underscore that infertility is associated with elevated levels of psychological distress in men (and women, though our focus is men). Men experiencing infertility report significantly higher rates of depressive symptoms, anxiety, and stress compared to fertile peers [78]. In a global review of literature from the past decade, researchers found consistently higher levels of depression and anxiety in individuals coping with infertility, especially those undergoing long and invasive treatments [78].

For men, infertility can strike at the core of self-identity and masculinity, leading to low self-esteem and social withdrawal. In fact, one study suggested that depression rather than anxiety has a more pronounced negative effect on semen quality in men, indicating a two-way relationship where mental health and fertility influence each other [79]. Men who were depressed had measurably poorer sperm parameters, possibly due to hormonal changes (like elevated cortisol or altered gonadotropin release) or health behaviors associated with depression (e.g., poor sleep, as that study noted men with depression and <7 h sleep had significantly reduced motile sperm counts) [79]. This finding is important because it implies that treating depression in infertile men might not only improve their quality of life but could also potentially improve their fertility outcomes.

Conversely, the stress of infertility itself can contribute to mental health problems that adversely affect physical health. Chronic stress and depression are known to increase inflammation, dysregulate the immune system, and exacerbate conditions such as hypertension and heart disease [80].

One particularly concerning outcome of severe psychological distress is suicidality. Although not commonly discussed, researchers in Sweden uncovered a slight but significant increase in suicide among men with infertility-related diagnoses [17]. Additionally, some infertile men may turn to maladaptive coping mechanisms, such as alcohol or substance abuse, which can obviously affect long-term health and mortality.

There is a growing expert consensus (2023–2025) that the evaluation of male infertility should be leveraged to improve overall men’s health. A recent European urology meta-analysis highlights male infertility as an “opportunity to improve preventive strategies for overall men’s health beyond the immediate reproductive goals”, reinforcing that psychosocial screening can be seamlessly integrated into broader preventive care [81].

It is important to recognize that not all infertile men will experience mental health issues–many cope resiliently, especially with strong partner support or counseling. However, given the prevalence of depression and anxiety in this group, healthcare providers are urged to screen infertile patients for psychosocial distress. Doing so is critical not only for improving their mental well-being but also because better mental health might lead to healthier behaviors and better engagement in medical care, thereby potentially improving physical health outcomes. While psychosocial factors such as depression and chronic stress often arise as secondary consequences of infertility, emerging evidence suggests they may also act as independent contributors to systemic health deterioration through neuroendocrine and inflammatory pathways [82].

## 6. Lifestyle, Socioeconomic Status, and Environmental Exposures

Lifestyle factors, socioeconomic determinants, and environmental exposures significantly influence both fertility and overall health. They likely act as common denominators that help explain why infertile men might have worse health outcomes. Some key considerations include.

### 6.1. Lifestyle Behaviors

Many habits and behaviors known to impair fertility also contribute to chronic disease [30]. Smoking is a prime example–it damages sperm DNA, reduces sperm count, and increases the risk of erectile dysfunction, while also being a leading cause of cardiovascular disease, respiratory illness, and cancers. An infertile man who smokes thus faces dual harm. Excessive alcohol consumption can lower testosterone and cause testicular atrophy, contributing to infertility; in the long run, alcohol abuse damages the liver, heart, and brain, shortening lifespan. Obesity and poor diet are well-recognized causes of subfertility: obesity alters testosterone/estrogen balance and increases oxidative stress in the testes [30]. The same obesity greatly raises risks of diabetes, heart disease, and early death. Sedentary lifestyle correlates with both erectile/fertility problems and metabolic illness. On the other hand, extreme exercise (endurance training) might temporarily reduce fertility by causing energy deficits and lower libido–though exercise in moderation generally benefits both fertility and health. Another relevant behavior is use of anabolic steroids or testosterone supplements by young men for bodybuilding–this can cause severe oligospermia or azoospermia (often reversible) and at the same time predispose to cardiac events and liver issues. Recreational drug use (e.g., marijuana, opiates) has also been linked to reduced sperm quality and sexual function, while clearly having systemic effects (like lung damage or addiction-related risks). Encouraging infertile men to adopt healthier lifestyles is a key intervention that could improve both their fertility odds and their long-term health prognosis.

### 6.2. Socioeconomic Determinants and Health Inequalities

The relationship between SES, fertility, and health is complex. On one hand, higher education and income often correlate with better health outcomes due to greater health literacy and access to care. On the other hand, infertility is diagnosed more often in higher-SES societies (where people have children later and seek medical help for childbearing). This could bias some cohorts of infertile men toward a population that is relatively advantaged, potentially underestimating their health risks when compared to the general population. Indeed, as noted, men evaluated in fertility clinics had lower mortality than the general population [12], presumably because they were a self-selected group with sufficient health awareness and resources. However, within cohorts of men seeking fertility care, those who are infertile still fare worse than fertile counterparts, indicating SES alone doesn’t negate the infertility-health link. Socioeconomic disadvantages (such as physically demanding or hazardous occupations, poor nutrition in childhood, limited healthcare access, high stress, and inability to afford treatments) could lead to untreated medical conditions that both impair fertility and shorten life. Moreover, not having children could influence economic security in old age (though this is more relevant in societies without social support systems). Overall, while SES might confound some observations, the consensus is that the health risks tied to male infertility are not solely explained by socioeconomic differences, yet clinicians should be attentive to the socioeconomic challenges infertile men might face, which can impact their health management.

Socioeconomic adversity further compounds the health challenges facing infertile men. Those of lower SES are disproportionately less likely to engage with healthcare or utilize fertility services, which can exacerbate long-term risks. For example, a U.S. survey found that infertile men who never accessed fertility care were more often of lower income, uninsured, and without a regular healthcare provider [83], suggesting many underlying conditions remain unaddressed. Likewise, subfertile men from deprived communities use fertility treatments at significantly lower rates and have reduced chances of live birth compared to those from affluent areas [84], reflecting disparities in access to care. The financial strain of infertility itself may further deteriorate health; nearly half of men pursuing fertility care report significant financial stress, a factor linked to worse health outcomes and higher mortality risk [85]. Thus, low SES can amplify the morbidity and mortality risks associated with male infertility by limiting medical intervention and adding chronic stress, even though infertility per se remains an independent health risk across all socioeconomic groups.

### 6.3. Environmental and Occupational Exposures

Over the last few decades, there has been concern about the role of environmental toxins in the decline of male reproductive health. Environmental exposures to certain chemicals and pollutants can simultaneously affect fertility and other health outcomes. For instance, endocrine-disrupting chemicals (EDCs)–such as phthalates (found in plastics), bisphenol A (BPA), certain pesticides, and industrial pollutants–have been shown to interfere with hormonal pathways. These compounds can reduce sperm counts and quality by disrupting the endocrine control of spermatogenesis [86,87]. At the same time, they may contribute to metabolic disorders and cancers; for example, some EDCs promote weight gain and insulin resistance [88], and others are implicated in increased risk of hormone-sensitive cancers. Heavy metal exposure (lead, mercury, cadmium) is known to impair spermatogenesis and also cause kidney, neurologic, or cardiovascular damage over time. Radiation (from extensive X-rays or radiation work) can mutate sperm and increase cancer risk. Occupational heat exposure (as experienced by welders or men who work in high-temperature environments) can lower sperm counts and might also indicate working conditions that predispose to dehydration or kidney disease. Thus, infertile men might be considered a sentinel population for environmental health hazards–if a toxin is impacting their fertility, it could very well be impacting their overall health.

### 6.4. The Role of Social and Relationship Factors

Lifestyle encompasses not just individual habits but also social context. Men who lack strong social support might not only experience more stress (affecting fertility) but also have worse health maintenance. Conversely, men in supportive relationships might adopt healthier lifestyles when trying to conceive (improving diet, reducing alcohol, etc.). However, if fertility treatments fail, some men might abandon these healthy changes. Social isolation, as discussed under psychosocial factors, can be both a cause and effect of infertility-related stress and is an established risk factor for morbidity (comparable to smoking in some analyses of mortality risk) [89].

**Figure 1:**
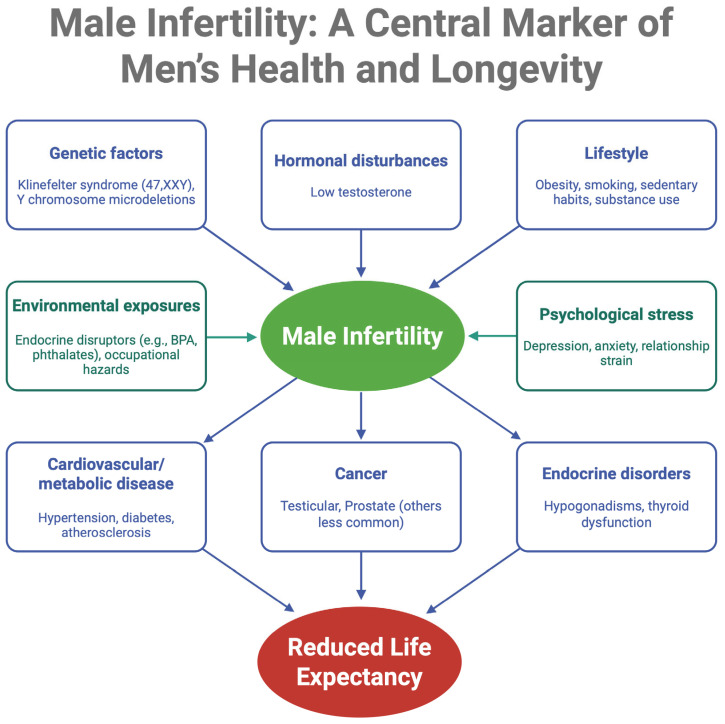
Male infertility as a central marker of men’s health and longevity: contributing factors and associated comorbidities.

The interconnected relationship between male infertility, associated risk factors, comorbid conditions, and ultimately reduced life expectancy is summarized in Figure 1, highlighting how infertility serves as a central marker of broader men’s health issues.

## 7. Clinical Implications and Recommendations

The recognition that infertile men have an elevated risk of various health problems and potentially shorter life expectancy carries important implications for clinical practice. A diagnosis of male infertility should prompt healthcare providers to think beyond conception–it is an opportunity to improve the man’s overall health trajectory. Key implications and recommendations include.

### 7.1. Comprehensive Health Screeing

Men presenting with infertility should receive a broad health assessment in addition to standard fertility testing. This includes screening for cardiovascular risk factors (checking blood pressure, fasting glucose, hemoglobin A1c [HbA1c], lipid profile), body mass index evaluation, and inquiry into lifestyle habits (smoking, alcohol, exercise, diet) [90]. As Eisenberg aptly stated, “As we treat men’s infertility, we should also assess their overall health”. A fertility clinic visit may be one of the few interactions a young man has with healthcare, so it’s a vital chance to catch silent conditions [91]. For example, discovering hypertension or prediabetes in an infertile man allows early intervention that could prevent a heart attack 10 years later. In practice, many fertility centers have begun to incorporate basic health screenings or refer men to primary care/urology for a complete physical [92]. Guidelines by urological associations increasingly emphasize that male infertility workups should include evaluation for underlying medical disorders [93].

### 7.2. Hormonal Evalutation and Management Strategies

Clinicians should have a low threshold to check hormonal profiles in infertile men–not only for fertility treatment (FSH, testosterone, etc.) but to identify conditions like hypogonadism, thyroid disease, or hyperprolactinemia that have wider health impacts. If an infertile man is found to have low testosterone, a nuanced approach is needed. In the short term, fertility and testosterone treatment are at odds (exogenous testosterone impairs sperm production) [93]. However, after family planning is complete or if sperm can be preserved, testosterone replacement therapy (TRT) might substantially improve the man’s metabolic health, bone density, and quality of life [94]. Recent data indicate that men who conceived via ICSI/IVF had a higher incidence of needing TRT in the years after (ICSI fathers had nearly 4-fold higher odds of starting TRT than natural conception fathers). This suggests many infertile men develop symptomatic hypogonadism relatively early [95]. Endocrinologists or urologists should monitor testosterone levels over time and treat as appropriate, balancing fertility desires. Additionally, addressing conditions like metabolic syndrome or diabetes with medications (e.g., metformin, statins) when indicated should be part of care–there is no reason to treat an infertile man differently from any other patient with those conditions, aside from attention to drug choices that don’t impair fertility [96].

Many infertile men have low testosterone, raising the question of whether this contributes directly to their elevated rates of chronic disease or merely reflects an underlying systemic issue. Cross-sectional evidence indicates that poor testicular function–characterized by low testosterone, elevated gonadotropins, and low sperm output–correlates with adverse metabolic and cardiovascular profiles [97]. For example, men with subnormal sperm counts have significantly higher odds of hypogonadism (≈12-fold in one large cohort) and tend to exhibit worse markers of cardiometabolic health [97]. This suggests that infertility and low testosterone often coincide as part of a broader health phenotype, although longitudinal studies are needed to clarify causality [97]. Hypogonadism is especially prevalent in men with severe spermatogenic failure (e.g., non-obstructive azoospermia), with studies reporting roughly 47–80% of such men meeting biochemical low-testosterone thresholds [26,98]. However, treating an infertile man’s hypogonadism poses a dilemma: while testosterone therapy can improve symptoms and metabolic parameters, exogenous testosterone suppresses the hypothalamic–pituitary–gonadal axis and drastically impairs spermatogenesis [99]. To avoid this, clinicians employ fertility-preserving hormonal strategies. Human chorionic gonadotropin (hCG) can stimulate the testes (Leydig cells) to produce testosterone endogenously, effectively raising serum levels without jeopardizing sperm production [100]. In hypogonadal men desiring fertility, hCG therapy–often supplemented with follicle-stimulating hormone (FSH) or human menopausal gonadotropin (hMG) to drive spermatogenesis–has proven successful, with the majority of cases achieving normal testosterone levels and restoring spermatogenesis (response rates ~85% in congenital hypogonadotropic hypogonadism) [100]. Furthermore, if a man has been on exogenous testosterone, discontinuation of therapy typically allows recovery of testicular function. Spermatogenesis usually resumes within months after stopping testosterone (with ~90% of men achieving a viable sperm count by ~12 months off treatment) [99], and endogenous testosterone production likewise returns toward baseline in most cases [100]. Accordingly, any testosterone-induced adverse effects (gonadotropin suppression, testicular atrophy, erythrocytosis, etc.) gradually reverse after cessation of therapy, though the benefits of testosterone on hypogonadal symptoms and risk factors may also wane once levels decline again.

### 7.3. Cancer Surveillance

Given the increased cancer risks, certain screening measures should be considered. All infertile men should undergo a thorough testicular exam, and those with a history of undescended testes or other risk factors might benefit from periodic ultrasonography to screen for testicular tumors. They should be educated about self-examination to catch testicular cancer early [101]. For prostate cancer, guidelines traditionally start screening at age 55 (or 45 if high risk), but an argument can be made for earlier and more frequent screening in men with severe male factor infertility [102]. The 2019 BMJ study authors suggested that men who used ICSI could be considered a high-risk group for early-onset prostate cancer [20]. While formal guidelines have not yet incorporated infertility as a risk factor for prostate cancer, some experts recommend a baseline PSA in the 40s for men with a history of idiopathic infertility or azoospermia [103,104]. Genetic testing is another facet: if an infertile man is found to have a genetic syndrome like Klinefelter or the carriers of BRCA (which can cause infertility and increase prostate cancer risk), then appropriate cancer surveillance (e.g., breast cancer screening for Klinefelter, early PSA for BRCA2 carriers) should be undertaken [105].

Population-based cohorts confirm an elevated incidence of testicular and prostate malignancies in previously infertile men, but the absolute risk increase is modest–lifetime occurrence remains well under 1% for both cancers [106]. Accordingly, no survival benefit from general early screening has been demonstrated, and infertility is not formally recognized as a risk factor warranting earlier routine prostate cancer screening in current guidelines [106]. Instead, expert consensus favors vigilant, risk-tailored surveillance: infertile men should undergo thorough testicular evaluation and be educated on regular self-examination (with adjunct ultrasound reserved for those who carry additional testicular cancer risk factors such as a history of cryptorchidism or intratesticular microcalcifications) [93]; likewise, some clinicians advocate a baseline PSA in the mid-40s for men with idiopathic infertility or azoospermia in light of data suggesting earlier PSA elevation in this group [103], even though definitive evidence of a mortality benefit is still lacking.

### 7.4. Intergrating Mental Health Support into Fertility Support

Integrating psychosocial support into infertility care is essential. Clinics should consider routine mental health screening questionnaires for depression and anxiety for men (and women) undergoing fertility evaluation. Counseling should be offered, and if needed, referral to a mental health professional experienced in dealing with infertility can be extremely beneficial [107]. Support groups or couples therapy can also help mitigate the emotional strain. By addressing mental health, providers not only help the patient cope better (which can improve treatment adherence and possibly even fertility outcomes) but also potentially reduce the risk of downstream issues like severe depression or suicidal ideation. It is also important to discuss the emotional aspects of alternative paths (such as IVF with donor gametes, adoption, or deciding to live childfree) in a supportive, non-judgmental way, because resolving the uncertainty and grief around infertility can allow men to move forward and focus on other aspects of life and health. Some studies have noted that when infertility is accepted or resolved, stress levels decrease, and health behaviors improve [108].

### 7.5. Lifestyle Interventions

As part of managing an infertile man, clinicians should strongly encourage and facilitate lifestyle modifications. This might include nutritional counseling for weight loss if the man is overweight, exercise programs, smoking cessation resources, and moderation of alcohol. Such interventions can have dual benefits: improving fertility parameters and reducing risk factors for chronic disease [30]. For example, weight loss in obese infertile men can raise testosterone and sperm counts and simultaneously improve blood pressure and glycemic control. Quitting smoking can improve erectile function (benefiting natural fertility potential) and reduce risks of cancer and heart disease. Because the motivation to conceive a healthy child can be a powerful incentive, fertility specialists can leverage this to promote positive behavior change [109]. Urologists and reproductive endocrinologists may coordinate with primary care or specialists (dietitians, etc.) to implement these changes. In some cases, treating a specific condition like varicocele may also slightly improve testosterone levels, which could be beneficial for metabolic health–so surgical or medical treatments for infertility could have broader health payoffs beyond aiding conception [110,111]. A recent meta-analysis further supports that varicocelectomy, FSH therapy, and lifestyle modifications may reduce sperm DNA fragmentation, with the most pronounced effect observed after six months post-varicocelectomy [112].

### 7.6. Long-Term Follow-up

After the immediate infertility issue is addressed, it is important that these men do not “fall off the radar.” Many couples, once they have a child or cease fertility treatments, might disengage from medical care. Healthcare providers should arrange ongoing follow-up for infertile men, preferably with a primary care physician who is informed of the potential health risks. This ensures continuity of care for managing blood pressure, cholesterol, blood sugar, hormone levels, etc. [25]. Some experts even propose developing a “male infertility survivor” care plan analogous to cancer survivorship–outlining what health checks and screenings an infertile man should undergo in the next decades of life [113].

Men who remain childless—a proxy for unresolved infertility—exhibit significantly higher rates of metabolic disorders (approximately 1.2-fold greater odds of metabolic syndrome and over 2-fold higher odds of type 2 diabetes) compared to fathers, as well as an elevated risk of cardiovascular mortality later in life [24]. Furthermore, the risk of all-cause mortality among infertile men rises with the severity of spermatogenic impairment, with the greatest hazards observed in those with azoospermia [114].

### 7.7. Patient Education and Counseling

Educating infertile men about the link between fertility and health is delicate but crucial. Many men may be unaware that their fertility issue could imply anything about their general health. When appropriate, physicians should explain that male infertility can sometimes be a marker of other health vulnerabilities [21]. This should be done in a reassuring way, emphasizing that being aware of these links is positive because it allows preventative action. This approach can frame health screenings as part of the fertility care, rather than an unrelated burden. Additionally, counseling about family planning options should go hand-in-hand with discussions about health. If a man’s infertility is due to an inheritable genetic condition, genetic counseling for his family is needed. If his condition could affect offspring health (e.g., DNA fragmentation, or older paternal age issues), that can also be addressed [115].

### 7.8. Interdisciplinary Collaboration

The multifaceted needs of infertile men call for collaboration between specialties: urologists/andrologists, endocrinologists, primary care physicians, cardiologists, psychologists, and others. For instance, a referral to an endocrinologist for managing newly diagnosed diabetes or thyroid disorder uncovered during infertility work-up could significantly benefit the patient. Likewise, coordinating with a cardiologist if severe hypertension is found, or a mental health therapist for coping strategies, will ensure comprehensive care. Reproductive medicine teams are increasingly adopting a multidisciplinary approach, sometimes including dedicated “men’s health” clinics that offer both fertility treatment and general health evaluation. This integrated care model is likely to yield the best outcomes in terms of both successful conception and long-term health [25].

### 7.9. Public Health Strategies and Awareness as a Window to Health

Recognizing that fertility status can serve as a “window into overall health” [116], experts have advocated for campaigns to educate men on how lifestyle and environmental exposures impact both fertility and long-term well-being. Such public health efforts target both the general population and healthcare providers, encouraging primary care physicians to seize any clinical encounter as an opportunity to address reproductive health and initiate basic fertility evaluations [117]. At the same time, international initiatives are bridging male reproductive health with preventive medicine: the EU-supported EcoFoodFertility project, for instance, utilizes semen quality as an early biomarker of environmental exposure impacts on human health [118], and the global Male Reproductive Health Initiative calls for greater education and engagement of men in their reproductive health across the lifespan [119]. By reframing male infertility as not only a fertility issue but also a general health concern, these strategies aim to destigmatize the topic and prompt earlier interventions, with the ultimate goal of improving men’s overall health [120].

## 8. Conclusions

Evidence increasingly suggests that male infertility is more than a reproductive concern: it is correlated with heightened risks of cardiovascular disease, metabolic dysfunction, and specific malignancies, as well as an overall increase in mortality. Genetic factors, hormonal imbalances—particularly hypogonadism—and potential lifestyle contributors appear to underlie this association. Moreover, the psychosocial impact of infertility, including stress, depression, and anxiety, can compound men’s vulnerability to chronic conditions. Male infertility thus emerges as a valuable “sentinel” or early warning sign, alerting healthcare providers to broader health issues that, if left unrecognized, may reduce life expectancy. Recognizing male infertility as a potential barometer of systemic health highlights the importance of comprehensive health screening, targeted counseling, and proactive management of comorbidities—ranging from cancer surveillance to cardiometabolic evaluations—during and after infertility treatment.

By shifting our perspective to view infertility as part of a man’s overall health profile, clinicians can turn a distressing diagnosis into an opportunity for preventive care and lasting wellness. Integrating mental health support, screening for endocrine and genetic abnormalities, and reinforcing lifestyle interventions, such as smoking cessation and weight management, are crucial steps. These measures not only bolster fertility outcomes but also mitigate long-term risks, including cancer, heart disease, and metabolic disorders. To further clarify the nature of these associations, prospective longitudinal studies are needed to determine whether infertility directly contributes to poorer health or reflects common underlying factors. Research into novel biomarkers will also help identify men at highest risk, guiding interventional trials to test whether targeted strategies—ranging from lifestyle modifications to pharmacological therapy—can improve both fertility and long-term health outcomes. As research into the mechanisms linking fertility and systemic health advances, incorporating male infertility evaluations into routine preventive medicine could significantly improve both the quality and length of men’s lives—yielding benefits that extend well beyond reproductive success.

## Figures and Tables

**Table 1:** Epidemiological evidence linking male infertility to increased mortality and cancer risks.

Study	Population Size (N)	Fertility Status	Mortality Risk (HR/RR)	Key Findings
Fallara et al. (2024) [15]	3,173,122 fertile vs. 212,791 infertile men	Infertile vs. fertile men	All-cause mortality: HR = 1.37 (95% CI: 1.04–1.81); Testicular cancer: RR = 1.86 (95% CI: 1.41–2.45); Prostate cancer: RR = 1.66 (95% CI: 1.06–2.61); Melanoma: RR = 1.30 (95% CI: 1.08–1.56); Diabetes: HR = 1.39 (95% CI: 1.09–1.71); Cardiovascular events: HR = 1.20 (95% CI: 1.00–1.44)	Infertile men had significantly increased risks of all-cause mortality, specific cancers (testicular, prostate, melanoma), diabetes, and cardiovascular events compared to fertile men, supporting infertility as a marker for general health risks.
Del Giudice et al. (2021) [14]	Total: 202,456 (infertility cohorts); 59,291 (semen parameters cohorts)	Infertile vs. fertile men; Oligo/Azoospermic vs. Normospermic	HR = 1.26 (95% CI: 1.01–1.59); RR = 1.67 (95% CI: 1.26–2.21) for Oligo/Azoospermic vs. Normospermic	Infertile men had a 26% higher all-cause mortality compared to fertile men. Those with combined oligo- and azoospermia had a 67% higher risk of death compared to normospermic men. Azoospermic men specifically had over twofold risk (HR = 2.17, 95% CI: 1.55–3.04) compared to normospermic controls.
Behboudi-Gandevani et al. (2021) [19]	168,327 infertile men; 2,252,806 controls	Infertility vs. Fertility	OR = 1.43 (95% CI 1.25–1.64)	Infertile men had approximately 43% higher risk of developing any cancer compared to fertile men. Specifically elevated risks were noted for testicular cancer (OR = 1.91, 95% CI: 1.52–2.42), prostate cancer (OR = 1.48, 95% CI: 1.05–2.08), and melanoma (OR = 1.31, 95% CI: 1.06–1.62). Male infertility was concluded to be a significant independent risk factor for future cancer development.
Elenkov et al. (2020) [18]	22,444 men from Malmö Preventive Project	Childless vs. Fathers (proxy for infertility)	HR (CVD mortality) = 1.33 (95% CI: 1.18–1.49); HR (all-cause mortality) = 1.23 (95% CI: 1.14–1.33)	Childless men exhibited significantly higher cardiovascular mortality (33% increased risk) and all-cause mortality (23% increased risk) compared to fathers. Childless men also had a worse baseline metabolic profile with increased odds of high triglycerides (OR 1.24), high fasting glucose (OR 1.23), and hypertension (OR 1.28). Suggests childlessness (likely related to infertility) independently predicts increased cardiovascular and metabolic risks.
Al-Jebari et al. (2019) [20]	1,181,490 fathers (20,618 IVF; 14,882 ICSI; 1,145,990 natural conception)	ICSI/IVF vs. Natural conception	HR = 1.64 (95% CI: 1.25–2.15) for ICSI; HR = 1.33 (95% CI: 1.06–1.66) for IVF; HR = 1.86 (95% CI: 1.25–2.77) for early-onset prostate cancer (ICSI)	Men fathering via ICSI had a 64% higher overall risk of prostate cancer compared with natural conception. ICSI-treated men had an 86% increased risk of early-onset prostate cancer (diagnosed before age 55). Fathers conceiving via IVF had a 33% higher risk of prostate cancer compared to natural conception. Severe male-factor infertility thus appears strongly associated with elevated long-term prostate cancer risks, particularly for early-onset disease.
Lundberg et al. (2019) [17]	43,598 men with infertility diagnosis; 57,733 men with infertility-related diagnosis; 2,762,254 controls	Infertility and infertility-related diagnoses vs. controls	HR = 0.98 (95% CI: 0.89–1.08) for infertility diagnosis; HR = 1.23 (95% CI: 1.17–1.30) for infertility-related diagnoses; HR = 3.26 (95% CI: 2.42–4.41) for mortality before age 30 in infertile men	Overall, men diagnosed with infertility did not have significantly higher mortality compared to controls. However, significantly higher mortality occurred among those with infertility-related diagnoses and particularly among infertile men under 30, largely due to cancers diagnosed before infertility evaluation. A slightly elevated suicide risk (HR = 1.18; 95% CI: 1.01–1.37) was also observed, indicating possible psychosocial implications.
Eisenberg et al. (2016) [16]	13,027 infertile men; 23,860 fertility-tested controls; 79,099 vasectomized men	Male infertility vs. fertility-tested and vasectomized men	Increased risk: Diabetes (HR 1.30, 95% CI 1.10–1.53), Ischemic heart disease (HR 1.48, 95% CI 1.19–1.84), Hypertension (HR 1.09, 95% CI 1.02–1.17), Renal disease (HR 1.60, 95% CI 1.14–2.24), Liver disease (HR 1.53, 95% CI 1.31–1.80), Peripheral vascular disease (HR 1.52, 95% CI 1.12–2.07)	Infertile men had significantly increased risks of developing chronic medical conditions including diabetes, ischemic heart disease, hypertension, renal and liver diseases, and peripheral vascular disease compared to fertile (vasectomized) men. Findings suggest infertility evaluation may identify men at increased risk for chronic health conditions later in life.
Eisenberg et al. (2014) [12]	11,935 infertile men	Normal vs. Abnormal semen parameters	HR = 2.29 (95% CI: 1.12–4.65) for men with ≥2 semen abnormalities	Over ~8 years follow-up, men with two or more abnormal semen parameters had more than double the risk of death compared to those with normal semen parameters. Study confirms that more severe sperm abnormalities predict higher risks of premature mortality.
Jensen et al. (2009) [11]	43,277 men	Normospermic vs. Oligo-/Azoospermic	Dose-response decrease in mortality with increasing sperm concentration (up to 40 million/mL), motility, and normal morphology (*p* < 0.05)	Men with higher sperm counts and greater percentages of motile and morphologically normal spermatozoa had significantly lower mortality rates. Mortality decreased steadily with improving semen quality parameters up to a concentration of 40 million/mL, supporting the concept of semen quality as a fundamental biomarker of overall male health and life expectancy.

HR = Hazard Ratio; RR = Relative Risk; OR = Odds Ratio; CI = Confidence Interval; ICSI = Intracytoplasmic Sperm Injection; IVF: In Vitro Fertilization; CVD: Cardiovascular Disease; vs.: versus; Ref. No.: Reference Number; ~: approximately; ≥: greater than or equal to; <: less than.

## Data Availability

No new data were created or analyzed in this study.
